# Supplementary material for: Unveiling the Bioactive Potential of the Invasive Jellyfish Phyllorhiza punctata Through Integrative Transcriptomic and Proteomic Analyses
Source: Biomolecules. 2025 Aug 4;15(8):1121. doi: 10.3390/biom15081121 (PMC12383608; doi:10.3390/biom15081121)
Supplement: Supplementary file 1 [file biomolecules-15-01121-s001.zip › Suplementary Material/FileS3/PEPFOLD4/XP_065071425_31/clusters.html]

 PEP-FOLD clusters


Download archive of all models  here   

|  |  |  |
| --- | --- | --- |
| # | file | sOPEP |
| # cluster 1 - Size: 4 |  |  |
|  | XP\_065071425\_31-model1 | -15.3973 |
|  | XP\_065071425\_31-model1.1 | -11.8339 |
|  | XP\_065071425\_31-model1.2 | -11.3061 |
|  | XP\_065071425\_31-model1.3 | -9.57557 |
| # cluster 2 - Size: 3 |  |  |
|  | XP\_065071425\_31-model2 | -15.0397 |
|  | XP\_065071425\_31-model2.1 | -13.7819 |
|  | XP\_065071425\_31-model2.2 | -11.9137 |
| # cluster 3 - Size: 1 |  |  |
|  | XP\_065071425\_31-model3 | -12.5443 |
| # cluster 4 - Size: 1 |  |  |
|  | XP\_065071425\_31-model4 | -12.1827 |
| # cluster 5 - Size: 3 |  |  |
|  | XP\_065071425\_31-model5 | -11.7558 |
|  | XP\_065071425\_31-model5.1 | -11.0426 |
|  | XP\_065071425\_31-model5.2 | -10.3866 |
| # cluster 6 - Size: 3 |  |  |
|  | XP\_065071425\_31-model6 | -11.7557 |
|  | XP\_065071425\_31-model6.1 | -10.2905 |
|  | XP\_065071425\_31-model6.2 | -7.50382 |
| # cluster 7 - Size: 2 |  |  |
|  | XP\_065071425\_31-model7 | -11.6688 |
|  | XP\_065071425\_31-model7.1 | -7.44483 |
| # cluster 8 - Size: 3 |  |  |
|  | XP\_065071425\_31-model8 | -10.6515 |
|  | XP\_065071425\_31-model8.1 | -6.80214 |
|  | XP\_065071425\_31-model8.2 | -6.44621 |
| # cluster 9 - Size: 2 |  |  |
|  | XP\_065071425\_31-model9 | -10.2547 |
|  | XP\_065071425\_31-model9.1 | -8.26708 |
| # cluster 10 - Size: 1 |  |  |
|  | XP\_065071425\_31-model10 | -9.7801 |
| # cluster 11 - Size: 3 |  |  |
|  | XP\_065071425\_31-model11 | -9.6904 |
|  | XP\_065071425\_31-model11.1 | -6.82867 |
|  | XP\_065071425\_31-model11.2 | -5.72191 |
| # cluster 12 - Size: 1 |  |  |
|  | XP\_065071425\_31-model12 | -9.30569 |
| # cluster 13 - Size: 2 |  |  |
|  | XP\_065071425\_31-model13 | -9.0035 |
|  | XP\_065071425\_31-model13.1 | -8.60236 |
| # cluster 14 - Size: 1 |  |  |
|  | XP\_065071425\_31-model14 | -8.90697 |
| # cluster 15 - Size: 5 |  |  |
|  | XP\_065071425\_31-model15 | -8.8918 |
|  | XP\_065071425\_31-model15.1 | -8.41673 |
|  | XP\_065071425\_31-model15.2 | -7.00563 |
|  | XP\_065071425\_31-model15.3 | -6.18121 |
|  | XP\_065071425\_31-model15.4 | -5.94192 |
| # cluster 16 - Size: 1 |  |  |
|  | XP\_065071425\_31-model16 | -8.86256 |
| # cluster 17 - Size: 10 |  |  |
|  | XP\_065071425\_31-model17 | -8.76252 |
|  | XP\_065071425\_31-model17.1 | -7.12661 |
|  | XP\_065071425\_31-model17.2 | -6.70379 |
|  | XP\_065071425\_31-model17.3 | -6.02051 |
|  | XP\_065071425\_31-model17.4 | -6.01288 |
|  | XP\_065071425\_31-model17.5 | -5.98173 |
|  | XP\_065071425\_31-model17.6 | -5.94167 |
|  | XP\_065071425\_31-model17.7 | -5.74363 |
|  | XP\_065071425\_31-model17.8 | -5.6904 |
|  | XP\_065071425\_31-model17.9 | -5.52852 |
| # cluster 18 - Size: 1 |  |  |
|  | XP\_065071425\_31-model18 | -8.73181 |
| # cluster 19 - Size: 2 |  |  |
|  | XP\_065071425\_31-model19 | -8.72676 |
|  | XP\_065071425\_31-model19.1 | -6.43509 |
| # cluster 20 - Size: 1 |  |  |
|  | XP\_065071425\_31-model20 | -8.50759 |
| # cluster 21 - Size: 1 |  |  |
|  | XP\_065071425\_31-model21 | -8.36869 |
| # cluster 22 - Size: 1 |  |  |
|  | XP\_065071425\_31-model22 | -8.28918 |
| # cluster 23 - Size: 2 |  |  |
|  | XP\_065071425\_31-model23 | -8.27457 |
|  | XP\_065071425\_31-model23.1 | -6.02574 |
| # cluster 24 - Size: 1 |  |  |
|  | XP\_065071425\_31-model24 | -8.24236 |
| # cluster 25 - Size: 1 |  |  |
|  | XP\_065071425\_31-model25 | -7.76687 |
| # cluster 26 - Size: 2 |  |  |
|  | XP\_065071425\_31-model26 | -7.64384 |
|  | XP\_065071425\_31-model26.1 | -6.06726 |
| # cluster 27 - Size: 4 |  |  |
|  | XP\_065071425\_31-model27 | -7.57833 |
|  | XP\_065071425\_31-model27.1 | -6.82617 |
|  | XP\_065071425\_31-model27.2 | -6.25807 |
|  | XP\_065071425\_31-model27.3 | -6.17751 |
| # cluster 28 - Size: 4 |  |  |
|  | XP\_065071425\_31-model28 | -7.54852 |
|  | XP\_065071425\_31-model28.1 | -6.51379 |
|  | XP\_065071425\_31-model28.2 | -5.90804 |
|  | XP\_065071425\_31-model28.3 | -5.85992 |
| # cluster 29 - Size: 1 |  |  |
|  | XP\_065071425\_31-model29 | -7.32018 |
| # cluster 30 - Size: 1 |  |  |
|  | XP\_065071425\_31-model30 | -7.17279 |
| # cluster 31 - Size: 4 |  |  |
|  | XP\_065071425\_31-model31 | -7.15227 |
|  | XP\_065071425\_31-model31.1 | -6.79727 |
|  | XP\_065071425\_31-model31.2 | -6.25138 |
|  | XP\_065071425\_31-model31.3 | -6.20702 |
| # cluster 32 - Size: 3 |  |  |
|  | XP\_065071425\_31-model32 | -7.14971 |
|  | XP\_065071425\_31-model32.1 | -6.36039 |
|  | XP\_065071425\_31-model32.2 | -5.65553 |
| # cluster 33 - Size: 2 |  |  |
|  | XP\_065071425\_31-model33 | -7.09122 |
|  | XP\_065071425\_31-model33.1 | -6.3939 |
| # cluster 34 - Size: 1 |  |  |
|  | XP\_065071425\_31-model34 | -6.87847 |
| # cluster 35 - Size: 2 |  |  |
|  | XP\_065071425\_31-model35 | -6.82905 |
|  | XP\_065071425\_31-model35.1 | -6.66694 |
| # cluster 36 - Size: 1 |  |  |
|  | XP\_065071425\_31-model36 | -6.77769 |
| # cluster 37 - Size: 2 |  |  |
|  | XP\_065071425\_31-model37 | -6.71122 |
|  | XP\_065071425\_31-model37.1 | -6.05478 |
| # cluster 38 - Size: 1 |  |  |
|  | XP\_065071425\_31-model38 | -6.59772 |
| # cluster 39 - Size: 2 |  |  |
|  | XP\_065071425\_31-model39 | -6.46043 |
|  | XP\_065071425\_31-model39.1 | -5.80786 |
| # cluster 40 - Size: 1 |  |  |
|  | XP\_065071425\_31-model40 | -6.38271 |
| # cluster 41 - Size: 1 |  |  |
|  | XP\_065071425\_31-model41 | -6.26332 |
| # cluster 42 - Size: 2 |  |  |
|  | XP\_065071425\_31-model42 | -6.11166 |
|  | XP\_065071425\_31-model42.1 | -5.69297 |
| # cluster 43 - Size: 1 |  |  |
|  | XP\_065071425\_31-model43 | -6.0467 |
| # cluster 44 - Size: 1 |  |  |
|  | XP\_065071425\_31-model44 | -6.0169 |
| # cluster 45 - Size: 1 |  |  |
|  | XP\_065071425\_31-model45 | -5.99057 |
| # cluster 46 - Size: 1 |  |  |
|  | XP\_065071425\_31-model46 | -5.90297 |
| # cluster 47 - Size: 1 |  |  |
|  | XP\_065071425\_31-model47 | -5.83519 |
| # cluster 48 - Size: 2 |  |  |
|  | XP\_065071425\_31-model48 | -5.81945 |
|  | XP\_065071425\_31-model48.1 | -5.54075 |
| # cluster 49 - Size: 1 |  |  |
|  | XP\_065071425\_31-model49 | -5.76273 |
| # cluster 50 - Size: 2 |  |  |
|  | XP\_065071425\_31-model50 | -5.66082 |
|  | XP\_065071425\_31-model50.1 | -5.55673 |
|  |  |  |
